# Design and Optimization of a BAW Magnetic Sensor Based on Magnetoelectric Coupling

**DOI:** 10.3390/mi13020206

**Published:** 2022-01-28

**Authors:** Wanchun Ren, Jintong Li, Guo Liu, Jiarong Chen, Si Chen, Zhijun Gu, Jianbo Li, Junru Li, Yang Gao

**Affiliations:** 1 School of Information Engineering, Southwest University of Science and Technology, Mianyang 621010, China; lijt@mails.swust.edu.cn (J.L.); chenjr@mails.swust.edu.cn (J.C.); chensi@mails.swust.edu.cn (S.C.); guzj@mails.swust.edu.cn (Z.G.); lijb@mails.swust.edu.cn (J.L.); 2 Robot Technology Used for Special Environment Key Laboratory of Sichuan Province, Mianyang 621010, China; 3 Science and Technology on Electronic Information Control Laboratory, Southwest China Research Institute of Electronic Equipment, Chengdu 610036, China; liuguosgg@hotmail.com; 4 College of Optoelectronic Engineering, Chongqing University, Chongqing 400044, China; li_junru@foxmail.com

**Keywords:** magnetic sensor, bulk acoustic wave, magnetic composite, ME heterostructure, resonance enhanced, magnetoelectric coupling

## Abstract

Magnetic sensors actuated by bulk acoustic wave (BAW) have attracted extensive attention due to the fact of their high sensitivity, GHz-level high frequency, and small size. Different from previous studies, suppression of energy loss and improvement in energy conversion efficiency of the BAW magnetoelectric (ME) sensor were systematically considered during the device design in this work. Finite element analysis models of material (magnetic composite), structure (ME heterostructure), and device (BAW ME magnetic sensor) were established and analyzed in COMSOL software. Additionally, the magnetic composite was prepared by radio frequency magnetron sputtering, and its soft magnetism was characterized by magnetic hysteresis loop and surface roughness. The research results demonstrate that after inserting four layers of 5 nm Al_2_O_3_ films, a performance of 86.7% eddy current loss suppression rate, a less than 1.1% magnetostriction degradation rate, and better soft magnetism were achieved in 600 nm FeGaB. Furthermore, compared with other structures, the two-layer piezomagnetic/piezoelectric heterostructure had a better ME coupling performance. Eventually, the design of the BAW ME magnetic sensor was optimized by the resonance-enhanced ME coupling to match the resonance frequency between the magnetic composite and the BAW resonator. When a 54,500 A/m direct current bias magnetic field was applied, the sensor worked at the first-order resonance frequency and showed good performance. Its linearity was better than 1.30%, the sensitivity was as high as 2.33 μmV/A, and the measurement range covered 0–5000 A/m.

## 1. Introduction

**Figure 1:**
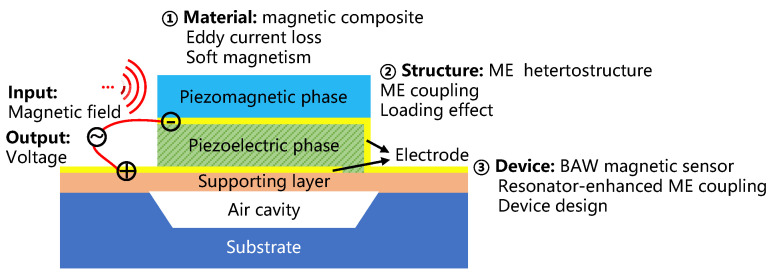
Mechanism of the BAW ME magnetic sensor and the optimizing method of the sensor’s performance by fine-tuning the design of the material, structure, and device to improve the energy coupling efficiency and decrease the energy loss.

Strong strain-mediated magnetoelectric (ME) coupling in magnetic/electric heterostructures have demonstrated good energy conversion between magnetic and electric fields. It shows great potential for practical devices such as sensors and tunable radio-frequency (RF)/microwave devices. The ME coupling effect is derived from the piezoelectric effect of the piezoelectric phase and the magnetostrictive effect of the piezomagnetic phase [1,2]. Generally, there are two types of ME heterostructures: bulk ME composites and thin-film ME heterostructures. Over the last two decades, many magnetic sensors with bulk ME composites have been reported; however, the device size was at the cm level or larger and difficult to reduce in size [3,4,5]. Compared to the bulk composites, micro-magnetic sensors based on thin-film ME heterostructures driven by acoustic waves have become a hot topic for their advantages of miniaturization, excellent elastic interactions, low cost, and the potential capability to integrate with conventional complementary metal oxide semiconductor (CMOS) technology [6,7,8,9]. Two types of magnetic sensors actuated by acoustic waves have been demonstrated: surface acoustic waves (SAWs) and bulk acoustic waves (BAWs). The sensor based on the SAW type is limited to working in the low- and medium-frequency band of kHz or measuring static/quasi-static magnetic field signals, although its static sensitivity is pretty high [10,11,12,13,14,15]. Whereas the sensor based on BAW excitation has attracted tremendous attention in recent years because of its high-frequency characteristics, high power capacity, and high energy conversion efficiency. The mechanism of the BAW ME magnetic sensor is shown in Figure 1; when the external RF magnetic field acts on the magnetostrictive material, strain occurs due to the magnetostrictive effect. Furthermore, because of the ME coupling between the piezomagnetic and piezoelectric phase, the strain transmitted to the piezoelectric material induces the longitudinal piezoelectric effect, resulting in positive and negative opposite charges on the opposite surface of the piezoelectric material, which are output by the electrode as a voltage. Especially, the ME coupling between the two types of films via elastic interaction becomes maximum at the mechanical resonant frequency of the heterostructures. Such resonance enhanced ME coupling greatly benefits the performance of the BAW ME magnetic sensor.

Much effort has been focused on the experimental and theoretical investigations of the BAW ME magnetic sensors. Hui et al. reported a MEMS resonant magnetic field sensor based on an AlN/FeGaB bilayer nano-plate resonator [16], ME coupling by depositing a composite ME heterostructure of a monolayer AlN/10 layers FeGaB/Al_2_O_3_ on an AlN CMR, and a ME structure based on nano-plate resonators was reported by Nan et al., which had good magnetic resolution [17,18]. Simultaneously, some works through modeling and simulation methods are also demonstrated. Wu et al. reported a flexible magnetic sensor based on a BAW resonator, the equivalent Mason model of the sensor circuit was established, and its sensitivity was improved by selecting the electrode of giant magnetostrictive material with a large frequency offset [19]. Martos et al. proposed a circuit simulation model of a novel miniaturized magnetoelectric antenna which is applied in low-power sensing [20]. However, there is little systematic research on the material, structure, and device simulation and performance optimization of the micro-magnetic sensor based on BAW actuation yet [21,22,23].

In order to design and optimize the BAW ME magnetic sensor, as shown in Figure 1, this paper proposes a method to decrease the eddy current loss of the magnetic composite and improve the energy coupling of different layers in the device by finite element analysis (FEA) simulation and experiments. Additionally, models of material (magnetic composite), structure (ME heterostructure), and device (BAW ME magnetic sensor) were established and analyzed. Meanwhile, the magnetic composite was prepared by the RF magnetron sputtering method and characterized to optimize its soft magnetism. Eventually, the design of a BAW ME magnetic sensor with higher sensitivity and better linearity can be achieved.

## 2. Materials and Methods

**Figure 2:**
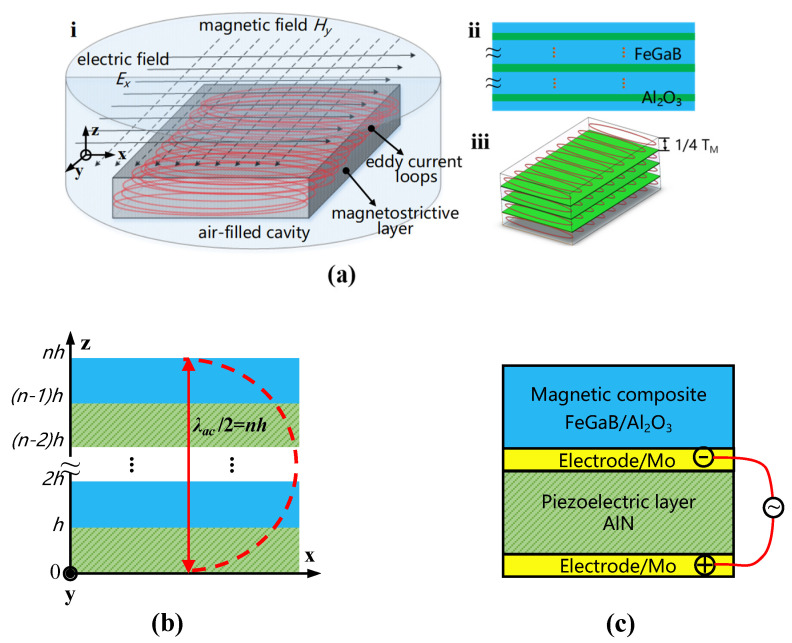
FEA models in COMSOL software. (**a**) Material: the magnetic composite, (**i**) a 3D model in an air-filled cavity for eddy current and magnetostriction simulation; (**ii**) magnetic composite—FeGaB inserted by alumina layers; (**iii**) suppression of eddy current loss—eddy current loops isolated by uniformly laminated alumina in FeGaB. (**b**) Structure: ME coupling effect of the piezomagnetic/piezoelectric heterostructure. (**c**) Device: resonance frequency matching between the FMR of the magnetic composite and the resonance frequency of the BAW resonator.

As shown in Figure 2a, a 3D simulation model of the magnetostrictive layer (FeGaB) was built in COMSOL Multiphysics software, and parameters of the films were referenced from data from Northeastern University. A magnetic field, *Hy*, and an electric field, *Ex*, were applied to the magnetostrictive layer in an air-filled cavity to generate high-frequency dynamic magnetic flux, which induced eddy current loops and magnetostriction in Figure 2a(i). The alumina films with different thickness (0~100 nm) and layers (0~10 pieces) were uniformly inserted into the magnetostrictive layer to form a magnetic composite in Figure 2a(ii), which induced a dramatic decrease in the eddy current density and somehow degradation of the magnetostriction in Figure 2a(iii) [24,25]. Furthermore, the thickness of the composite film was optimized in the range of 0~3000 nm. In order to characterize the soft magnetism of the magnetic composite, FeGaB (600 nm) and (FeGaB (120 nm)/Al_2_O_3_ (5 nm))_4_/FeGaB (120 nm) were layer by layer deposited on the SiO_2_ substrates with 100 Oe bias field by an RF magnetron sputtering tool at the frequency of 13.56 MHz, there was no vacuum broken between the FeGaB and Al_2_O_3_ deposition. The sputtering power was RF 80 W for FeGaB and RF 90 W for Al_2_O_3_, while the sputtering pressure and base pressure were 0.7 and 4 × 10^−4^ Pa, respectively. The cross-section and diffraction pattern were characterized by TEM (transmission electron microscope) and EDX (energy-dispersive X-ray spectroscopy). Magnetic hysteresis loops and surface roughness were tested by VSM (vibrating sample magnetometer) and AFM (atomic force microscope), respectively. Furthermore, the permeability of the magnetic composite was measured by ESR (electron spin resonance).

As shown in Figure 2b, the structure based on the 2–5 layer ME heterostructure was simulated in the COMSOL Multiphysics software [6]. The FEA models of the ME heterostructure, including the piezomagnetic phase, piezoelectric phase, and air domain, were constructed by coupling the magnetic field, solid mechanics module, and electrostatic module in the 3D geometric model. The strain, ME coefficient, and voltage were simulated and compared under the conditions of DC bias to optimize the structure of the ME heterostructure.

As shown in Figure 2c, based on the optimization result of the material and structure, an FEA model of the magnetic sensor was constructed to analyze resonance enhanced ME coupling between ferromagnetic resonance (FMR) of the magnetic composite and resonance frequency of the resonator by fine-tuning the device size. Eventually, the design of the BAW ME magnetic sensor was finalized, and the sensitivity, linearity, and full scope of the sensor were optimized.

## 3. Results and Discussion

### 3.1. Material Design: Magnetic Composite

#### 3.1.1. Eddy Current Loss

**Figure 3:**
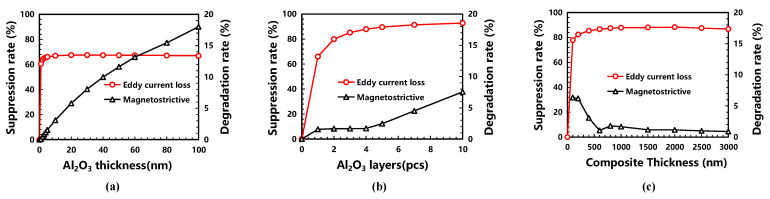
Suppression of the eddy current loss and degradation of magnetostriction by inserting an alumina insulation layer into the FeGaB: (**a**) the thickness effect of the single-layer alumina; (**b**) the layer number effect of the inserted 5 nm alumina; (**c**) the thickness effect of the magnetic composite.

As shown in Figure 3a, the suppression rate of the ECL increased sharply with the increase in Al_2_O_3_ thickness; then, it reached its saturation value at a thickness of 10 nm. In particular, at a thickness of 5 nm, the suppression rate reached 98.5% of the saturation value, but its overall suppression value was not higher than 66.8%. Therefore, the effective ECL suppression could not be achieved yet just by increasing the thickness of the Al_2_O_3_ film. At the same time, the degradation rate of the strain tensor kept increasing almost linearly with the increase in the thickness of the Al_2_O_3_ film. After inserting a single 5 nm Al_2_O_3_ film, the degradation rate of strain tensor could be controlled within 1.6% and obtained a 66.8% saturated suppression rate of ECL.

Multi-layer Al_2_O_3_ films were inserted into the magnetic material, and a significant improvement in the suppression rate can be observed in Figure 3b. The increasing rate of ECL suppression gradually slowed down and then reached the maximum of 95.6%. To insert multi-layer Al_2_O_3_ with a thickness of 5 nm, a higher suppression rate of over 90% can be achieved, because the inserted insulation film can separate the eddy current loop into several weaker ones by limiting the eddy current within a narrower space. Simultaneously, the magnetostriction degradation rate of the magnetic film increased slowly with the increase in the number of layers. It was less than 1.8% with 1~4 layers of inserted Al_2_O_3_ film; then, it increased near-linearly beyond four layers, reaching 7.8% for 10 layers. Therefore, with a trade-off between magnetostriction and ECL suppression, inserting four layers of 5 nm Al_2_O_3_ films into 1000 nm FeGaB film, the degradation rate of the magnetostriction was less than 1.8%, and the ECL suppression reached more than 85.1%.

As shown in Figure 3c, with the increase in composite thickness, the suppression rate changed into the shape of a “rainbow”, which is supposed to be caused by the interaction of the isolation effect and size effect. These two effects alternately dominate before and after the thickness of 800 nm, respectively. Moreover, less degradation of magnetostriction for thicker magnetic composites, especially at an ultralow 1.1% magnetostriction degradation and 86.7% ECL suppression of the composites, was found at a thickness of 600 nm, which may be enhanced by well magnetic coupling between two laminated magnetostrictive layers after introducing a certain number of interfaces.

#### 3.1.2. Soft Magnetism

**Figure 4:**
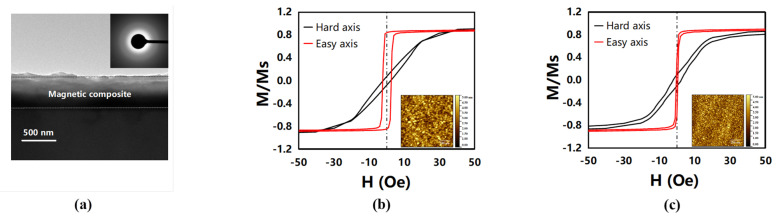
Soft magnetism of the magnetic composite: (**a**) the cross-section and diffraction pattern of (FeGaB (120 nm)/Al_2_O_3_ (5 nm))_4_/FeGaB (120 nm) by TEM and a comparison of the soft magnetism between (**b**) FeGaB and (**c**) (FeGaB (120 nm)/Al_2_O_3_ (5 nm))_4_/FeGaB (120 nm) through a magnetic hysteresis loop by VSM and surface roughness by AFM.

As depicted in Figure 4a, the magnetic composite showed an amorphous state by the cross-section and diffraction pattern, which can effectively suppress the crystallization and grain growth of the film. Therefore, the soft magnetism of the composite can be enhanced by decreasing the magnetocrystalline anisotropy and raising the inter-grain exchanging coupling [25]. In Figure 4b,c, the magnetic hysteresis loops and surface roughness were measured to compare the soft magnetism between the FeGaB film and the composite, and a 98.9% and 35.2% decrease in the coercivity and surface roughness, respectively, were achieved after inserting the four-layer alumina into the FeGaB film. Magnetostatic interaction and surface roughness were also considered to play positive roles in improving soft magnetic properties.

### 3.2. Structure Design: ME Heterostructure

Under different bias magnetic fields, the energy coupling of the ME heterostructure was analyzed through the energy conversion of the magneto–electro–mechanical. Its coupling generated an induced charge on the surface of the piezoelectric layer, resulting in an induced voltage. Therefore, the structure of the sensor can be optimized by comparing the strain, ME coefficient, and the output voltage of the ME heterostructures in different layers. The strain and ME coefficient, α_ME_, are the most important parameters to evaluate the coupling performance of the ME heterostructure. Their calculation formulas are as following equations [26]:

(1) $$S_{H}=\frac{1}{2}[(\nabla u{)}^{T}+\nabla u]$$

(2) $$\alpha_{ME}=\frac{\partial E_{z}}{\partial H_{\mathrm{bias}}}=\frac{\partial E_{z}}{\partial H_{y}}$$

As shown in Equation (1), S_H_, T, and **u** are the strain tensor, stress tensor, and displacement of the magnetostrictive layer, respectively. The strain of the magnetostrictive layer varies with its displacement gradient. Therefore, the ME coupling effect of the piezomagnetic/piezoelectric heterostructure is mediated by strain or mechanical energy. As shown in Equation (2), E_z_ is the electric field added along the z-direction, and the DC bias, H_bias_, is the magnetic field added along the y-direction, H_y_. The ME coefficient can be used to characterize the ME coupling efficiency of heterostructures.

**Figure 5:**
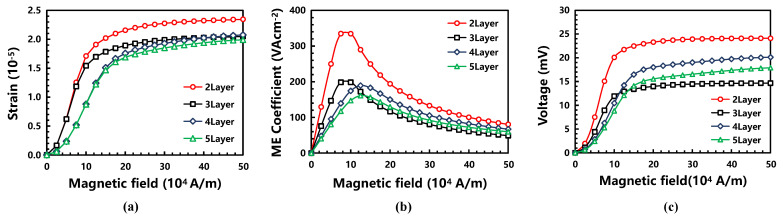
With the increase in the bias DC magnetic field: (**a**) the variation curve of the strain, (**b**) the variation curve of the ME coefficient, and (**c**) the output voltage of the ME heterostructures in different layers.

As shown in Figure 5a, the strain in the two-layer structure was the largest. This is because the strain of the magnetostrictive layer was affected by the displacement gradient (Equation (1)), and its strain change law was consistent with that of the displacement gradient. Furthermore, the variation law of the ME coefficient was analyzed under the bias magnetic field (0~500 Oe) (Equation (2)). The ME coefficient firstly increased and then decreased with the addition of the bias magnetic field. The two-layer ME heterostructure with the highest ME coefficient had the highest sensitivity, which also means the best magnetoelectric conversion efficiency and the largest output voltage. As shown in Figure 5c, the output voltage value of the two-layer structure was the largest one, too. Therefore, the structure based on the two-layer ME heterostructure can achieve the best energy coupling.

In summary, the result demonstrates a two-layer structure for improving the sensitivity of the magnetic sensor by optimizing its strain, ME coefficient, and the output voltage of the ME heterostructure layers.

### 3.3. Device Design: BAW Magnetic Sensor

**Figure 6:**
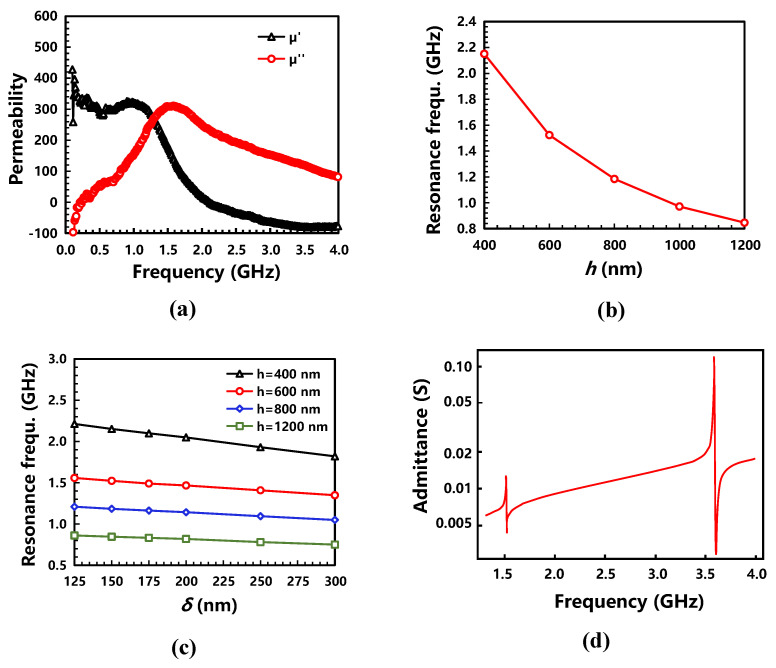
Design of the magnetic sensor by resonance enhanced ME coupling: (**a**) permeability of the FeGaB/Al_2_O_3_ composite by ESR and the FMR matching by changing the thickness of (**b**) the piezomagnetic/piezoelectric heterostructure and (**c**) the electrode; (**d**) after matching the frequency, the admittance curve of the two-layer device was obtained by applying an AC voltage to the piezoelectric layer of the ME heterostructure without a bias magnetic field.

The permeability of the magnetic composite is shown in Figure 6a, and its resonance frequency was 1.51 GHz. In order to obtain the highest output voltage by resonance enhanced ME coupling, the BAW ME magnetic sensor should work at the same resonance frequency [27]. Therefore, the device size, including the thickness of the piezoelectric/magnetic layer and the electrode, needs to be optimized to match the FMR of the magnetic composite and the resonance frequency of the BAW resonator. Figure 6b,c show that the resonance frequency of the sensor can be fine-tuned by adjusting the thickness of the ME heterostructure and the electrode. As shown in Figure 6d, the resonance frequency matched well between the BAW resonator (2.65 GHz) and films (piezoelectric 600 nm/magnetic 600 nm; Mo electrode 200 nm) by considering the mass load effect. The first-order and second-order resonance frequencies of the sensor were 1.51 and 3.60 GHz, respectively.

### 3.4. Performance Analysis

**Figure 7:**
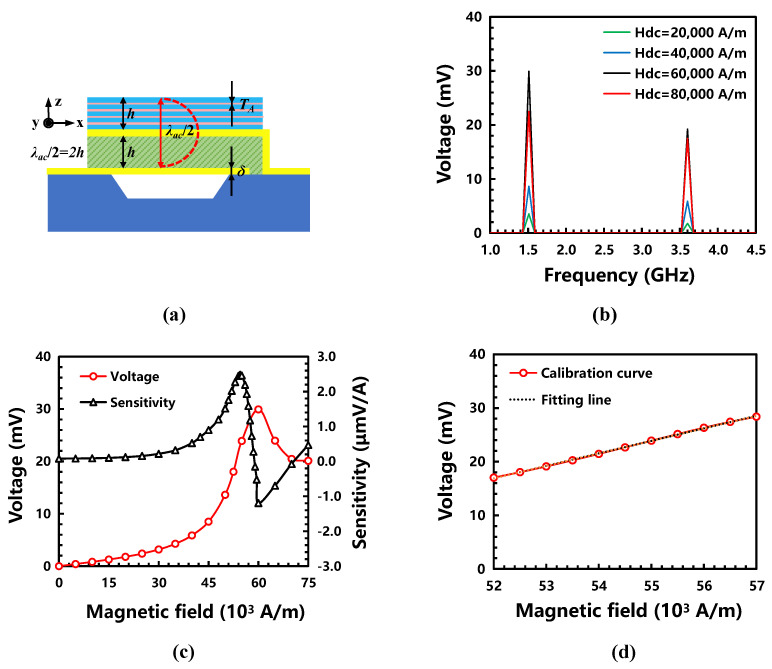
The performance of the BAW ME magnetic sensor: (**a**) the finalized design of the BAW ME magnetic sensor; (**b**) the output voltage variation at the first-order and the second-order resonance frequencies; (**c**) the output voltage and sensitivity at the first-order resonance frequency under different DC bias magnetic fields; (**d**) the linearity and the measurement range with a DC bias of 54,500 A/m.

As shown in Figure 7a, the design of the BAW ME magnetic sensor was optimized, including the magnetic composite (FeGaB with a four-layer alumina uniformly inserted; T_A_ was 5 nm), the structure (two-layer piezomagnetic/piezoelectric heterostructure; h was 600 nm), and the electrode (*δ* was 200 nm). In order to evaluate the performances of the magnetic sensor, different DC biases magnetic fields (20,000–80,000 A/m) were applied to characterize the output voltage and ME coefficients (Figure 7b). The output voltage at the resonance frequency was significantly higher than that of others; additionally, the maximum output voltage at the first-order resonance frequency was larger than that of the second-order one.

The sensitivity and linearity were the most important parameters to evaluate the BAW ME magnetic sensor. Their calculation formulas are shown in the following equations:

(3) $$S=\frac{\partial V}{\partial H_{y}}=\frac{\partial E_{z}}{\partial H_{y}}h$$

(4) $$\alpha_{L}=\frac{\Delta Y_{\max}}{Y_{\mathrm{FS}}}\times 100\%$$

As shown in Equation (3), S, V, and h are the sensitivity, output voltage, and thickness of the piezoelectric phase, respectively. It was found that the sensitivity of the sensor was dependent on the ME coupling of piezomagnetic/piezoelectric heterostructure, α_ME_, and thickness of piezoelectric phase, h. In order to obtain good ME coupling, it can thus be seen that it is extremely necessary to improve energy conversion efficiency and suppress eddy current loss in the design of the BAW ME magnetic sensor. In Equation (4), a_L_, ∆*Y*_max_, and *Y*_FS_ are the linearity, the maximum deviation between the calibration curve and the fitting line, and the output voltage difference over the full-scale range. Therefore, the linearity of the sensor was directly related to a selection of the fitting line and a full-scale range. In this work, the fitting line was obtained by the least squares method. In Figure 7c, it can be found that the output voltage at the first-order resonance frequency increased to be the highest and then decreased at the bias magnetic field of 60,000 A/m; meanwhile, the sensitivity at the DC bias magnetic field of 54,500 A/m was found to be the highest by the differential analysis method of the *V–H* curve. As shown in Figure 7d, a high sensitivity of 2.33 μmV/A, a good linearity better than 1.30%, and a wide measurement range of 0–5000 A/m could be achieved while the DC bias magnetic field applied on the sensor was kept at 54,500 A/m.

## 4. Conclusions

In this work, a method was proposed to design and optimize the BAW ME magnetic sensor; especially, the energy loss suppression and the energy conversion efficiency improvement were systematically considered. FEA models of material (magnetic composite), structure (ME heterostructure), and device (BAW ME magnetic sensor) were established and analyzed in COMSOL software. Additionally, the magnetic composite was prepared by RF magnetron sputtering, and its soft magnetism was characterized by magnetic hysteresis loop and surface roughness.

After inserting four-layers of 5 nm Al_2_O_3_ films, the performance of an 86.7% eddy current loss suppression rate, less 1.1% magnetostriction degradation rate, and smaller coercivity and roughness were achieved for (FeGaB (120 nm)/Al_2_O_3_ (5 nm))_4_/FeGaB (120 nm). Furthermore, the strain, ME coefficient, and output voltage of the heterostructure were simulated and analyzed. Compared with other structures, the two-layer piezomagnetic/piezoelectric heterostructure had a better ME coupling performance. Eventually, the design of the BAW ME magnetic sensor was optimized by matching the resonance frequency between the magnetic composite and the BAW resonator as the first-order of 1.51 GHz. When a 54,500 A/m DC bias magnetic field was applied, the sensor worked at the first-order resonance frequency and showed good performance. The linearity was better than 1.30%, the sensitivity was as high as 2.33 μmV/A, and the measurement range could cover 0–5000 A/m. With the advantages of the highest energy conversion efficiency based on resonance enhanced ME coupling and the lowest eddy current loss and the integrated capability with CMOS technology, the BAW ME magnetic sensor has a bright future for compact receiving antennas, biomedical application, and the Internet of Things (IoT) due to the fact of its unique and particular properties. Furthermore, this achievement will further guide the structural design and performance optimization of other ME coupling devices.
